# Adverse prognosis of epigenetic inactivation in *RUNX3* gene at 1p36 in human pancreatic cancer

**DOI:** 10.1038/sj.bjc.6604333

**Published:** 2008-05-13

**Authors:** S Nomoto, T Kinoshita, T Mori, K Kato, H Sugimoto, N Kanazumi, S Takeda, A Nakao

**Affiliations:** 1 Department of Surgery II, Graduate School & Faculty of Medicine, University of Nagoya, Nagoya, Japan

**Keywords:** *RUNX3*, pancreatic cancer, hypermethylation, loss of heterozygosity

## Abstract

Alteration in transforming growth factor-*β* signalling pathway is one of the main causes of pancreatic cancer. The human runt-related transcription factor 3 gene (*RUNX3*) is an important component of this pathway. *RUNX3* locus 1p36 is commonly deleted in a variety of human cancers, including pancreatic cancer. Therefore, we examined genetic and epigenetic alterations of *RUNX3* in human pancreatic cancer. Thirty-two patients with pancreatic cancer were investigated in this study. We examined the methylation status of *RUNX3* promoter region, loss of heterozygosity (LOH) at 1p36, and conducted a mutation analysis. The results were compared with clinicopathological data. Promoter hypermethylation was detected in 20 (62.5%) of 32 pancreatic cancer tissues, confirmed by sequence of bisulphite-treated DNA. Loss of heterozygosity was detected in 11 (34.3%) of 32 pancreatic cancers. In comparison with clinicopathological data, hypermethylation showed a relation with a worse prognosis (*P*=0.0143). Hypermethylation and LOH appear to be common mechanisms for inactivation of *RUNX3* in pancreatic cancer. Therefore, *RUNX3* may be an important tumour suppressor gene related to pancreatic cancer.

Despite its relatively low incidence of approximately 10 cases/100 000 people, pancreatic cancer is still one of the leading causes of cancer-related death in industrialised countries including Japan. The prognosis remains poor, with an overall 5-year survival rate of less than 5% (Jemal *et al*, 2007). The pathogenesis of pancreatic ductal adenocarcinoma can be described as a step-by-step accumulation of genetic changes, such as K-ras oncogene mutations, p53, p16, and smad4 tumour suppressor gene mutations (Kern *et al*, 2002), in addition to several epigenetic alterations, which together result in self sufficiency of growth signals, insensitivity to antigrowth signals, evasion of apoptosis, angiogenesis, invasion, and metastasis (Ozawa *et al*, 2001). Recently, several reports indicated that every silencing mechanism, such as loss of heterozygosity (LOH) and mutations in a gene, or hypermethylation in its promoter region occurred in a tumour suppressor gene resulting in loss of its function in tumorigenesis (Tokumaru *et al*, 2003).

Transforming growth factor-*β* (TGF-*β*) signalling is a well-established tumour suppressor pathway in pancreatic carcinogenesis (Massagu *et al*, 2000). Smad4 is a key transcription factor in the TGF-*β*1 signalling pathway, and is inactivated in about 50% of pancreatic adenocarcinomas. The human runt-related transcriptional factor 3 (*RUNX3*) gene also plays important roles in the TGF-*β* signalling pathway. In this pathway, Smad2 and Smad3 activated by TGF-*β* interact with RUNX3, and induce transcriptional activation of target genes in the nucleus (Ito and Miyazono, 2003; Miyazono *et al*, 2003).

RUNX3 induced apoptosis in epithelial cells, and the knockout mice of this gene showed hyperplasia in gastric mucosa. In addition, loss of function of RUNX3 caused by DNA hypermethylation, LOH at gene locus, and mutation correlated with the progression of primary gastric cancers (Li *et al*, 2002). RUNX3 might have the important role of TGF-*β* and Smad proteins in carcinogenesis. Furthermore, *RUNX3* is located on the distal portion of the short arm of human chromosome 1 (1p36), which is commonly deleted in a variety of human cancers, including pancreatic cancer (Nowak *et al*, 2005; Loukopoulos *et al*, 2007). Therefore, the genetic and epigenetic alterations in *RUNX3* may have an important role in pancreatic cancer.

The aim of our present study was to determine whether the *RUNX3* gene alteration might have a role in carcinogenesis in pancreatic cancer. We examined LOH at this gene locus in 1p36 with microdissected DNA, the DNA-methylation status by methylation-specific polymerase chain reaction (MSP) and sequencing, and the mutation of *RUNX3* by reverse transcription-polymerase chain reaction (RT-PCR) single-strand conformation polymorphism (RT-PCR-SSCP) in 32 primary pancreatic cancer tissues and corresponding noncancerous tissues. Then, we correlated these results with the clinicopathological data.

## MATERIALS AND METHODS

### Patients, sample collection, microdissection, and DNA preparation

Thirty-two primary pancreatic cancer tissues and corresponding noncancerous tissues were collected at Nagoya University Hospital from pancreatic cancer patients during pancreatico-duodenectomy, distal pancreatectomy, or total pancreatectomy. All tissues were diagnosed histologically as pancreatic cancer. Written informed consent, as required by the institutional review board, was obtained from all patients. Collected samples were stored immediately in liquid nitrogen at −80°C until analysis. Genomic DNA was obtained from these samples by digestion with proteinase K, followed by phenol/chloroform extraction.

Other parts of the specimens were formalin-fixed for 24 h and processed for paraffin embedding. From each tissue block, a series of four 5-*μ*m thick sections were cut. The first section was H&E stained for pathologic evaluation; identification of the tumour epithelia. To avoid normal cell contamination, target epithelial cells from the cancer areas were produced by laser capture microdissection using a Pixcell LCM system (Arcturus Engineering Inc., Mountain View, CA, USA). An average of 200 laser shots (30 *μ*m shot size, 60 ms laser pulse duration, and power of 60 MW) were used for each sample. Microdissected cells were then incubated overnight at 37°C in 50 *μ*l digestion buffer (10 mmol l^−1^ Tris-HCl (pH 8.0), 1 mmol l^−1^ EDTA, 1% Tween 20, 1 mg ml^−1^ proteinase K) and incubated at 95°C for 10 min to inactivate the proteinase K.

### Microsatellite analysis

DNAs from primary pancreatic cancer tissues and corresponding noncancerous tissues were analysed for LOH study by amplification of CA repeat sequences using PCR. DNAs of pancreatic cancer epithelia were collected by the microdissection method mentioned above.

Two microsatellite markers, D1S234 and D1S247, were used. D1S234 exists at only 900 Kb on the telomeric side from the RUNX3 locus, and D1S247 is centromeric from the *RUNX3* locus. Polymerase chain reaction amplification was performed containing [*α*^32^P]dCTP and 50 ng of genomic DNA. Polymerase chain reaction products were analysed on a 6% polyacrylamide gel and processed by autoradiography. Allelic loss was scored when the band intensity of one allele was decreased significantly (more than 40% reduction) in tumour DNA as compared with that in the normal DNA by using a BASS-2000 image analyzer (Fuji Photo Film Co. Ltd, Tokyo, Japan).

### Methylation-specific PCR

DNA from tumour and normal specimens was subjected to bisulphite treatment. Briefly, 2 *μ*g of DNA was denatured by NaOH and modified by sodium bisulphite. DNA samples were then purified using the Wizard purification resin (Promega Corp., Madison, WI, USA), treated again with NaOH, precipitated with ethanol, and resuspended in water. The primer pairs for the unmethylated detecting were in *RUNX3* promoter region near exon 1: S (sense, 5′-GTGGGTGGTTGTTGGGTTAGT-3′) and AS (antisense, 5′-TCCTCAACCACCACTACCACA-3′), which amplify a 138-base pair (bp) product, and those for the methylated detecting were in the same region: S (sense, 5′-CGTCGGGTTAGCGAGGTTTC-3′) and AS (antisense, 5′-GCCGCTACCGCGAAAAACGA-3′), which amplify a 120-bp product. The PCR amplification consisted of 35 cycles of 94°C for 20 s, 60°C for 20 s, and 72°C for 15 s, after the initial denaturation step (94°C for 5 min). Each PCR product was loaded directly onto 2% agarose gels, stained with ethidium bromide, and visualised under UV illumination.

### Sequence analysis

Genomic bisulphite-treated DNA of primary pancreatic cancer tissues was sequenced. Polymerase chain reaction was performed in methylated cases. The primer pairs for sequence were in RUNX3 promoter region near exon1: S (sense, 5′-GTTTAGGTAGTAGGGATAGTT-3′) and AS (antisense, 5′-CTATTCTCTCCCATCTTACC-3′), which amplify a 388-bp product. The PCR amplification consisted of 36 cycles of 94°C for 30 s, 54°C for 30 s, and 72°C for 30 s, after the initial denaturation step (94°C for 5 min). Polymerase chain reaction products were purified directly using the QIA quick Gel Extraction Kit (QIAGEN, Hilden, Germany). Purified DNA fragments were subcloned into TA cloning vector (Invitrogen™, Carlsbad, CA, USA). Six cloning samples were picked out from one methylated tumour tissue. Each cloning DNA was mixed with 3 *μ*l of specific primer (M13), 4 *μ*l of Cycle Sequence Mix (ABI PRISM Terminator v1.1 Cycle Sequencing Kit; Applied Biosystems, Foster City, CA, USA). Samples were subjected to the following cycling conditions: 95°C for 30 s; 25 cycles of 95°C for 15 s, 50°C for 15 s, and 60°C for 4 min followed by purification by ethanol precipitation. Sequence analysis was carried out using an Applied Biosystems ABI310, and sequence electropherograms were generated by ABI Sequence Analysis 3.0.

### RT-PCR-SSCP

Polymerase chain reaction amplification using random-primed cDNA of 32 primary pancreatic cancer tissues was performed using oligonucleotide primers in the presence of [*α*^32^P]dCTP, followed by electrophoretic separation on 6% nondenaturing polyacrylamide gels both in the presence of 5% glycerol at room temperature and in its absence at 4°C. RUNX3 ORF (1248-bp) is divided into four overlapped fragments and each fragment was amplified. The primer pairs used for *RUNX3* mutation were S1 (sense, 5′-GCCGCTGTTATGCGTATTCC-3′) and AS1 (antisense, 5′-CTCAGCGGAGTAGTTCTCGT-3′), amplifying a 370-bp fragment; S2 (sense, 5′-GTGACTGTGATGGCAGGCAA-3′) and AS2 (antisense, 5′-GTTCCGAGGTGCCTTGGATT-3′), amplifying a 398-bp fragment; S3 (sense, 5′-ACAAGCCACTTCAGCAGCCA-3′) and AS3 (antisense, 5-GAGAACTGGTAGGAGCCAGA-3′), amplifying a 368-bp fragment; S4 (sense, 5′-CTACCACCTCTACTACGGGA-3′) and AS4 (antisense, 5′-CCCATCACTGGTCTTGAAGG-3′), amplifying a 326-bp fragment. The PCR amplification consisted of 35 cycles of 94°C for 30 s, 58°C for 30 s, and 72°C for 30 s, after the initial denaturation step (94°C for 5 min) in F1–R1 and in the presence of 10% dimethylsulphoxide (F2–R2, F3–R3, F4–R4).

### Statistical analysis

The correlation between the methylation status of RUNX3 mRNA and clinicopathological data was analysed by Fisher's exact test or *χ*^2^ test for independence. Overall survival rates were calculated using the Kaplan–Meier method, and difference in survival curves was analysed using the log-rank test. Independent prognostic factors were identified by multivariate analysis using the Cox proportional hazards regression model. Data are expressed as mean±s.d. Statistical significance was considered as *P*<0.05.

## RESULTS

### Microsatellite analysis of *RUNX3*

**Figure 1:**
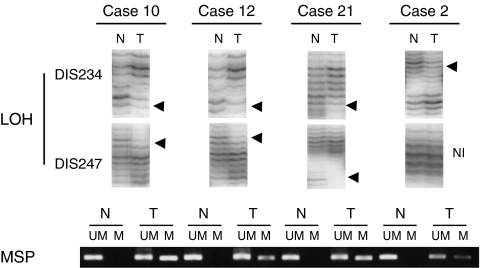
Representative results of LOH and MSP in cases 10, 12, 21, and 2. In the analysis of LOH at RUNX3 locus, cases 10, 12, and 21 showed allelic imbalance at D1S234 as well as at D1S247 (arrowheads). Case 2 showed allelic imbalance at D1S234 (arrowhead), but the D1S247 was not informative (NI). Promoter hypermethylation was observed in the DNA extracted from tumour tissue (T). In noncancerous samples (N), a methylation band was not seen in any lane. All four cases showed both LOH and promoter hypermethylation. These results indicated that biallelic inactivation (LOH at 1p36+ methylation) caused the inactivation of RUNX3 in pancreatic cancer. LOH, loss of heterozygosity; MSP=methylation-specific PCR; RUNX3=human runt-related transcription factor 3 gene.

We first examined DNA samples obtained by microdissection from the 32 primary pancreatic cancer tissues and corresponding noncancerous tissues for LOH using two microsatellite markers, D1S234 and D1S247, which are close to the RUNX3 locus. D1S234 is telomeric and D1S247 is centromeric to the locus. Allelic imbalance in one or two markers was observed in 11 (34.3%) of the 32 cases (Figure 1). We judged the 11 cases as having an LOH at the locus. The results are summarised in Table 1. No cases evidenced microsatellite instability in this study. Two cases proved noninformative from using the two markers.

### Hypermethylation of *RUNX3* promoter region in pancreatic cancer

**Figure 2:**
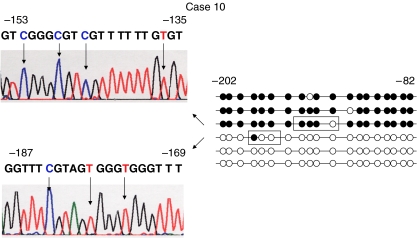
Sequence analysis of bisulphite-treated DNA from tumour sample of case 10 in RUNX3 promoter region. Methylation status of the 19 CpG islands between −82 and −202 from the transcription-initiation site of RUNX3 exon 1 is shown. The fragment was PCR amplified and subcloned into TA cloning vector. Closed circle indicates methylated CpG island, open circle indicates unmethylated CpG island. Each group of six clones showed a different methylation status. Arrows below the sequence indicate CpG islands. The Cs indicate methylated CpG islands. The Ts were converted from C by bisulphite treatment, indicating unmethylated CpG islands. RUNX3=human runt-related transcription factor 3 gene.

To investigate whether the gene silencing was due to hypermethylation of *RUNX3*, MSP was performed in the 32 primary pancreatic cancer tissues and corresponding noncancerous tissues. Promoter hypermethylation was detected in 20 (62.5%) of the 32 primary pancreatic cancer tissues and in only two of the corresponding noncancerous tissues (Figure 1). To confirm the methylation of the RUNX3 promoter region, genomic bisulphite-treated DNA of primary pancreatic cancer tissues, which showed methylation by MSP, were sequenced. Every case showed at least one methylated CpG island of the sequenced fragments. A representative case is shown in Figure 2.

### Mutational analysis of *RUNX3* in pancreatic cancer tissues

**Figure 3:**
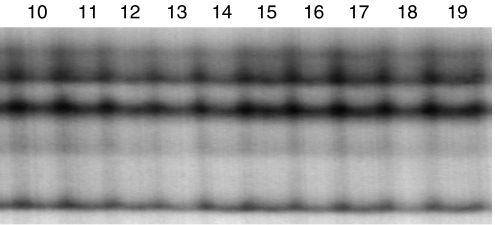
RT-PCR-SSCP analysis of RUNX3 in pancreatic cancer tissues. Representative results (cases 10–19) of RT-PCR-SSCP analysis using F2–R2 primer set. There were no aberrant bands in all cases. RT-PCR-SSCP=RT-PCR single-strand conformation polymorphism; RUNX3=human runt-related transcription factor 3 gene.

To investigate the mutation status of this gene, RT-PCR-SSCP analysis was performed. We could not see any aberrant bands (Figure 3). No mutations or polymorphisms were detected in the 32 pancreatic cancer tissues. As we used the bulk frozen samples, normal cells such as fibrosis cells were contaminated in the tumour tissues, making it difficult to identify aberrant bands.

### Statistical analysis of clinicopathological data and our findings

**Figure 4:**
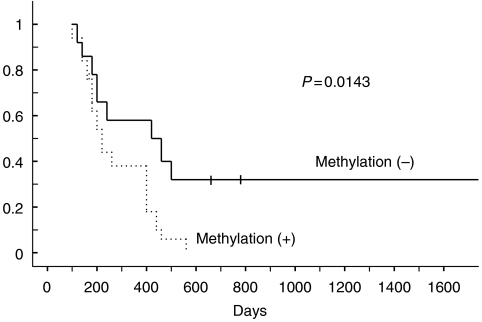
Survival stratified by methylation status in primary pancreatic cancer. RUNX3 hypermethylation was significantly correlated with a worse prognosis (*P*=0.0143). RUNX3=human runt-related transcription factor 3 gene.

Subsequently, we analysed the correlation between the clinicopathological data and results of our findings. Table 2 shows the correlation between the clinicopathological data and methylation status. Interestingly, *RUNX3* hypermethylation was significantly correlated with a worse prognosis (*P*=0.0143) (Figure 4). No other correlation with any clinicopathological parameter was found.

To evaluate the value of *RUNX3* methylation as an independent prognostic determinant, multivariate analysis was performed with prognostic factors that had been found to be significant by univariate analyses. The analysis identified lymph node metastasis, invasion of retroperitoneal tissue, and hypermethylation of *RUNX3* gene as the variables for independently predicting overall survival (Table 3).

## DISCUSSION

Transforming growth factor-*β* plays a key role in regulating the growth and differentiation of many cell types. In TGF-*β*1-null animals, proliferation of the gastric epithelium is stimulated and hyperplasia occurs (Crawford *et al*, 1998). TGF-*β* is known to be a potent inhibitor of pancreatic acinar and duct cell proliferation *in vitro* (Bisgaard and Thorgeirsson, 1991; Logsdon *et al*, 1992). RUNX3 is a runt domain transcription factor involved in this signalling pathway. RUNX3 protein binds with the Smad2 and Smad3 proteins. Recently, it has been reported that RUNX3 was one of the tumour suppressor genes in gastric cancer and testicular yolk sac tumour. Runx3-null mice reportedly develop hyperplasia of the gastric mucosa through activation of cellular proliferation and suppression of apoptosis in epithelial cells (Li *et al*, 2002). Interestingly, 1p36, where *RUNX3* exists, is a region commonly deleted in a wide variety of human carcinomas, including pancreatic cancer. To date, there are many reports regarding the TGF-*β* signalling pathway in pancreatic cancer (e.g. TGF-*β* receptor II, Smad2 and Smad4), but only a few deal with this gene’s alterations in pancreatic cancer (Li *et al*, 2004; Wada *et al*, 2004). Moreover, there are no reports regarding primary pancreatic cancer. Our study further supports a role for RUNX3 in pancreatic cancer.

The 1p36 region is believed to harbour tumour suppressor genes, because previous studies identified frequent allelic imbalance at 1p36 in various types of human cancers (Schwab *et al*, 1996). *RIZ1* and p73 genes are located on 1p36, and LOH was detected at each gene locus in pancreatic cancer (Sakurada *et al*, 2001; Sphyris *et al*, 2004). It is thought that these are one of the tumour suppressor genes in pancreatic cancer, and we think that RUNX3 may also be a candidate.

Previously, Wada *et al* (2004) reported that nine of 12 pancreatic cancer cell lines exhibited no expression of *RUNX3* by both northern blot analysis and RT-PCR. All of the nine cell lines showed methylation of the promoter CpG island of the gene. Moreover, hemizygous deletion of *RUNX3*, as detected by fluorescence *in situ* hybridisation, was found in most of the cell lines that lacked RUNX3 expression. Our results using primary pancreatic cancer tissue were compatible with their findings.

Li *et al* (2004) reported that RUNX3 expression was low-to-absent in normal pancreatic tissues, but increased in a third of cancer tissues by RT-PCR and immunohistochemistry. RUNX3 expression was present only in islets of the normal pancreas. They also found that all metastases of pancreatic cancer tissues were devoid of or displayed only very faint RUNX3 expression by immunostaining.

Some groups have advocated islet cells as the cells of origin of pancreatic ductal adenocarcinoma (Pour *et al*, 2003). This would mean that the islet cells in pancreatic tissue are the tissue-specific stem cells in which cancer cells begin from the alteration in the oncogenes or tumour suppressor genes. RUNX3 is expressed in the tissue-specific stem cells, and only in islet cells in normal tissue. When cancer tissue has grown from the tissue-specific stem cells, the cancer cells express the RUNX3 protein. Some cancer tissues do not express RUNX3. In those cancer cells, RUNX3 gene is methylated. In cases with metastatic lesions, more aggressive tumour cells from the original lesion exist, such as RUNX3-methylated cells. Hence, the metastatic pancreatic cancer cells do not express RUNX3 gene.

Thus, it may be hypothesised that there is indeed loss of RUNX3 expression by promoter hypermethylation or LOH in some primary tumours compared with normal islets, and almost a complete loss in metastatic tumours. Our finding that the survival in methylated cases in *RUNX3* gene was significantly worse than that in unmethylated patients is compatible with this hypothesis, although pointing to a tumour suppressor role for RUNX3 in pancreatic cancer.

Nine of 11 LOH detected cases had hypermethylation of the RUNX3 promoter region. These findings imply that silencing of RUNX3 occurred biallelically. Complete silencing of this gene leads to the progression of cancer, and then relates to the worse prognosis.

In conclusion, we have clearly demonstrated for the first time that *RUNX3* is frequently methylated in primary pancreatic cancer tissues, frequent hemizygous deletion occurs at its locus in 1p36, and RUNX3-inactivated cases showed worse survival. We propose that inactivation of RUNX3 plays an important role in alteration of the TGF-*β* signalling pathway and in the tumorigenesis of pancreatic cancer.

## Figures and Tables

**Table 1:** Clinicopathological features and results of RUNX3 alterations in pancreatic cancer tissues

					**Hypermethylation**		**LOH^a^**	
**Case**	**Gender**	**Location**	**Stage^b^**	**Pathology**	**N**	**T**	**D1S234**	**D1S247**
1	M	Ph	III	Tub. poor	—	—	•	•
2	F	Ph	IVa	Tub. mod	—	—	○	NI
3	M	Ph	IVb	Tub. mod	—	M	•	•
4	F	Ph	III	Tub. mod	—	—	•	NI
5	F	Phb	III	Anap. duc	—	—	NI	•
6	F	Ph	IVa	Tub. well	M	M	•	•
7	M	Ph	III	Tub	—	M	NI	•
8	F	Ph	IVb	Tub	—	M	•	•
9	M	Ph	IVb	Tub. mod	—	M	○	•
10	F	Ph	IVa	Tub. mod	—	M	○	○
11	M	Ph	IVb	Tub. mod	M	M	○	NI
12	M	Pb	III	Tub. poor	—	M	○	O
13	F	Ph	IVa	Tub. mod	—	—	•	•
14	M	Ph	IVa	Tub. mod	—	M	NI	•
15	M	Ph	IVb	Tub. mod	—	—	NI	•
16	M	Ph	IVb	Tub. poor	—	M	•	•
17	M	Ph	IVb	Tub. mod	—	—	•	○
18	M	Ph	IVb	Tub. mod	—	M	○	NI
19	M	Ph	IVb	Undifferentiated	—	M	•	○
20	F	Ph	IVb	Tub. mod	—	—	•	•
21	F	Phbt	IVb	Tub. mod	—	M	•	○
22	M	Ph	III	Acinar cell ca.	—	M	○	○
23	F	Ph	IVb	Tub	—	—	•	•
24	F	Phb	IVa	Tub. mod	—	—	NI	NI
25	F	Ph	IVa	Tub. poor	—	M	NI	•
26	M	Pb	IVa	Tub. well	—	M	NI	NI
27	F	Ph	III	Tub. mod	—	M	•	NI
28	M	Ph	IVa	Tub. mod	—	M	•	NI
29	M	Ph	III	Tub. mod	—	—	•	NI
30	M	Ph	IVa	Tub. well	—	—	•	NI
31	M	Pb	IVa	Tub. poor	—	M	NI	•
32	F	Ph	IVa	Tub. mod	—	M	○	○
					2/32 (6.3%)	20/32 (62.5%)	8/32 (25%)	7/32 (21.9%)
							LOH^*^: 11/32 (34.3%)	

a Anap. duc=anaplastic ductal adenocarcinoma; F=female; LOH=loss of heterozygosity; M=male; M=methylated; mod=moderately differentiated adenocarcinoma; N=normal tissue; NI=not informative; Pb=pancreatic body; Pt=pancreatic tail; poor=poorly differentiated adenocarcinoma; Ph=pancreatic head; T=tumour tissue; tub=tubular adenocarcinoma; well=well-differentiated adenocarcinoma; ; —, unmethylated; open circle=LOH detected; closed circle=retention of heterozygosity; LOH^*^=cases in which LOH was detected in at least one locus.

b The stage classification was performed according to the Pancreatic Cancer Study Group of Japan.

**Table 2:** Clinicopathological features and results of RUNX3 hypermethylation in pancreatic cancer tissues

		**Hypermethylation**		
**Variable**	**No. of cases**	+	−	***P*** **a**
*Age*				
<60	10	5	5	0.325
⩾60	22	15	7	
				
*Gender*				
M	18	13	5	0.198
F	14	7	7	
				
*Tumour size*				
TS1	5	2	3	>0.9999
⩾TS2	27	18	9	
				
*S*				
−	18	11	7	0.854
+	14	9	5	
				
*RP*				
−	10	6	4	0.844
+	22	14	8	
				
*CH*				
−	13	9	4	0.515
+	19	11	8	
				
*DU*				
−	21	13	8	0.923
+	11	7	4	
				
*PV*				
−	12	8	4	0.706
+	20	12	8	
				
*A*				
−	27	16	11	0.379
+	5	4	1	
				
*PL*				
−	27	16	11	0.379
+	5	4	1	
				
*DPM*				
−	26	15	11	0.242
+	6	5	1	
				
*N*				
0	14	8	6	0.581
1	18	12	6	
				
*Differentiation*				
Mod	21	12	9	0.241
Poor	6	5	1	

a Analysed by Fisher's exact test or *χ*^2^ test for independence.

^b^Tumour size according to the Classification of Pancreatic Carcinoma; A=arterial invasion; CH=choledocal invasion; DPM=dissected peripancreatic tissue margin; DU=duodenal invasion; F=female; PL=peripancreatic nerve plexus invasion; M=male; mod=moderately differentiated adenocarcinoma; N=lymph node metastasis; poor=poorly differentiated adenocarcinoma; pTNM=pathological TNM; PV=portal vein invasion; RP=retroperitoneal invasion; S=serosal invasion.

^c^Classified according to the classification of The General Rules for the Clinical and Pathological Study of Primary Pancreatic Cancer. April 2002, Pancreatic Cancer Study Group of Japan.

**Table 3:** Multivariate analysis of patients with pancreatic cancer

**Variable**	**Odds ratio**	**95% CI**	***P***
Tumour size (⩾2.0 cm)	1.995	0.639–6.226	0.2342
Lymph node metastasis	2.388	1.026–5.561	0.0435^*^
Invasion of retroperitoneal tissue (d.p.m.)	5.486	1.409–21.358	0.0141^*^
Invasion of plexus nerve (Pl)	1.759	0.591–5.239	0.3103
Hypermethylation	3.157	1.226–8.130	0.0172^*^

^*^Statistical significance.

CI=confidence interval.
